# Supplementary material for: Splice-Junction-Based Mapping of Alternative Isoforms in the Human Proteome
Source: Cell Rep. Author manuscript; Available in PMC 2020 Jan 15. (PMC6961840; doi:10.1016/j.celrep.2019.11.026)
Supplement: 3 [file NIHMS1546469-supplement-3.zip › DF2/PXD009737/TestisTrypsin-470-Q8WXG6-VYLYEGLLGK.pdf]

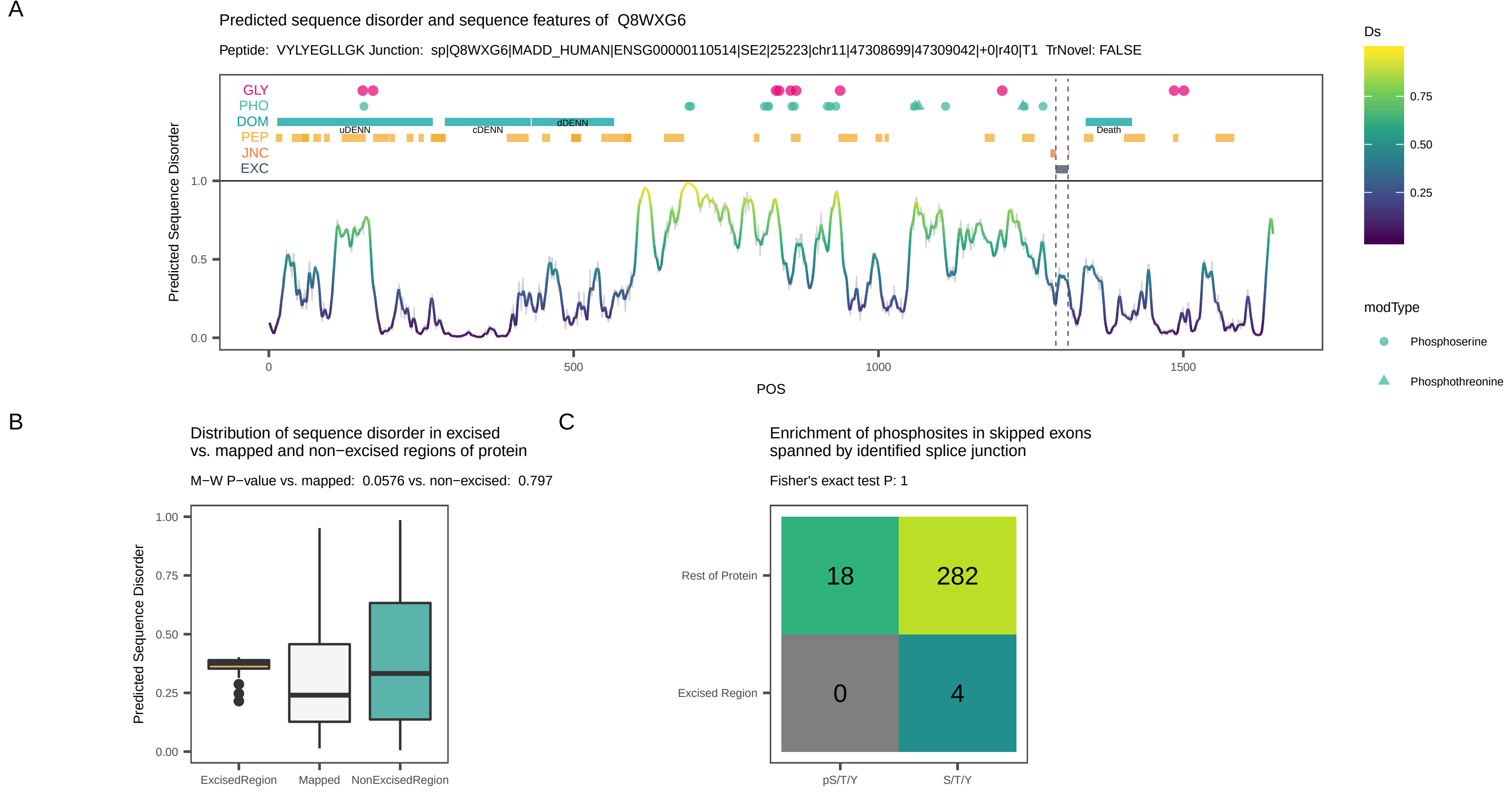
A
Predicted sequence disorder and sequence features of Q8WXG6
Peptide: VYLYEGLLGK Junction: sp|Q8WXG6|MADD_HUMAN|ENSG00000110514|SE2|25223|chr11|47308699|47309042|+0|r40|T1 TrNovel: FALSE
GLY
PHO
DOM
PEP
JNC
EXC
uDENN
cDENN
dDENN
Death
Predicted Sequence Disorder
1.0
0.5
0.0
0
500
1000
1500
POS
Ds
0.75
0.50
0.25
modType
Phosphoserine
Phosphothreonine
B
Distribution of sequence disorder in excised
vs. mapped and non−excised regions of protein
M−W P−value vs. mapped: 0.0576 vs. non−excised: 0.797
Predicted Sequence Disorder
1.00
0.75
0.50
0.25
0.00
ExcisedRegion
Mapped
NonExcisedRegion
C
Enrichment of phosphosites in skipped exons
spanned by identified splice junction
Fisher's exact test P: 1
Rest of Protein
Excised Region
18
282
0
4
pS/T/Y
S/T/Y
